# MR-guided focused ultrasound for painful bone metastases: safety when combined with chemotherapy

**DOI:** 10.1186/2050-5736-3-S1-O50

**Published:** 2015-06-30

**Authors:** Joshua Meyer, Raphael Pfeffer, Sergey Kanaev, Dmitri Iozeffi, David Gianfelice, Pejman Ghanouni, Daniela Militianu, Mark Hurwitz

**Affiliations:** 1 Fox Chase Cancer Center, Philadelphia, Pennsylvania, United States; 2 Assuta Hospital, Tel Aviv, Israel; 3 N.N. Petrov Research Institute of Oncology, Saint Petersburg, Russian Federation; 4 Rostov Scientific Research Institute of Oncology, Rostov-On-Don, Russian Federation; 5 Laennec Imagix Imaging Center • Medical Imaging, Ville Mont-Royal, Canada; 6 Stanford University, Stanford, California, United States; 7 Rambam Health Care Campus, Haifa, Israel; 8 Jefferson University Hospitals, Philadelphia, Pennsylvania, United States

## Background/introduction

Magnetic Resonance guided Focused Ultrasound (MRgFUS) is a non-invasive, non-ionizing treatment producing thermal ablation using high intensity focused ultrasound and MR thermometry to denervate pain from bone metastases. A recent phase III study comparing MRgFUS to sham treatment for a painful bone metastasis allowed patients to be treated with concurrent chemotherapy as long as the regimen was stable for at least 4 weeks. We performed a retrospective analysis of the safety of combination MRgFUS with active systemic chemotherapy in patients treated on this study.

## Methods

Chemotherapy data were available for 104 patients who were treated with MRgFUS in 17 medical centers worldwide as part of a randomized phase III study comparing MRgFUS to sham treatment for a painful bone metastasis previously irradiated or unsuitable for radiation therapy. Patients were followed for 3 months. Response was defined as a combination of at least two-point decrease on a standard numerical rating scale and <25% increase from baseline in analgesic medication at 3 months follow-up. Toxicity data was collected as any adverse event possibly related to treatment or its associated procedures. In spite of being an expected part of the procedure, sonication pain was scored as an event. Patients initially randomized to sham treatment who were offered rescue MRgFUS were also included in this analysis, bringing the safety population to 121 patients. All comparisons were performed using a z-score, with a significance level of 0.05.

## Results and conclusions

Ninety patients were treated without chemotherapy, and fourteen were treated with chemotherapy. There was no significant difference between the response rates of the chemotherapy group (71%) and the non-chemotherapy group (68%) (p=0.78). The overall event rates were 57% for chemotherapy patients and 45% for non-chemotherapy patients (p=0.38). Sonication pain was not significantly different between the groups, with 50% pain in the chemotherapy group and 28% pain in the non-chemotherapy group (p=0.11). Other events were limited to one patient with numbness of the skin in the chemotherapy group (7%). The non-chemotherapy group reported 18 events that were not sonication pain (17%). These rates were not significantly different (p=0.17). In this retrospective analysis of a prospective study, no difference was found in efficacy or toxicity of MRgFUS between patients receiving and not receiving active chemotherapy. Further studies are necessary to confirm the safety of these two modalities in combination. If confirmed, this may be an important reason to choose MRgFUS in the palliation of pain from bone metastases.

